# Influence of passive leg movements on blood circulation on the tilt table in healthy adults

**DOI:** 10.1186/1743-0003-1-4

**Published:** 2004-10-25

**Authors:** David Czell, Reinhard Schreier, Rüdiger Rupp, Stephen Eberhard, Gery Colombo, Volker Dietz

**Affiliations:** 1 Spinal Cord Injury Center, Balgrist University Hospital, Zurich, Switzerland; 2 Orthopaedic Hospital of Heidelberg University, Department II, Heidelberg, Germany; 3 Hocoma AG, Medical engineering, Volketswil, Switzerland

**Keywords:** tilt stepper, tilt table, blood circulation, syncope, near-syncope, vertical mobilization

## Abstract

**Background:**

One problem in the mobilization of patients with neurological diseases, such as spinal cord injury, is the circulatory collapse that occurs while changing from supine to vertical position because of the missing venous pump due to paralyzed leg muscles. Therefore, a tilt table with integrated stepping device (tilt stepper) was developed, which allows passive stepping movements for performing locomotion training in an early state of rehabilitation. The aim of this pilot study was to investigate if passive stepping and cycling movements of the legs during tilt table training could stabilize blood circulation and prevent neurally-mediated syncope in healthy young adults.

**Methods:**

In the first experiment, healthy subjects were tested on a traditional tilt table. Subjects who had a syncope or near-syncope in this condition underwent a second trial on the tilt stepper. In the second experiment, a group of healthy subjects was investigated on a traditional tilt table, the second group on the tilt ergometer, a device that allows cycling movements during tilt table training. We used the chi-square test to compare the occurrence of near-syncope/syncope in both groups (tilt table/tilt stepper and tilt table/tilt ergometer) and ANOVA to compare the blood pressure and heart rate between the groups at the four time intervals (supine, at 2 minutes, at 6 minutes and end of head-up tilt).

**Results:**

Separate chi-square tests performed for each experiment showed significant differences in the occurrence of near syncope or syncope based on the device used. Comparison of the two groups (tilt stepper/ tilt table) in experiment one (ANOVA) showed that blood pressure was significantly higher at the end of head-up tilt on the tilt stepper and on the tilt table there was a greater increase in heart rate (2 minutes after head-up tilt). Comparison of the two groups (tilt ergometer/tilt table) in experiment 2 (ANOVA) showed that blood pressure was significantly higher on the tilt ergometer at the end of head-up tilt and on the tilt table the increase in heart rate was significantly larger (at 6 min and end of head-up tilt).

**Conclusions:**

Stabilization of blood circulation and prevention of benign syncope can be achieved by passive leg movement during a tilt table test in healthy adults.

## Background

Several studies have confirmed that lack of movement leads quickly to profound negative physiological and biochemical changes in all organs and systems of the body [1-5]. It is important for patients suffering from diseases such as stroke, spinal cord and traumatic brain injury to be mobilized at an early state of rehabilitation [6]. As these patients are bedridden, their lower limbs are mainly mobilized through manual therapy or with cycling ergometers. Patients with spinal cord injuries are disposed to the occurrence of circulatory collapse when changing from a horizontal to a vertical position because of the lack of sympathetic activity and the missing contractions of leg muscles in the lower extremities that normally act as muscle pumps [7,8]. This instability of the circulatory system occurs at an early stage of rehabilitation and leads to delayed functional training of these patients. In a chronic phase, an overactivity of the spinal sympathetic system could take place, which can lead to vasoconstriction and hypertension [9].

Head-up tilt table testing has been used for over 50 years by physiologists and physicians for many purposes. This includes the study of the human body's heart rate and blood pressure adaptations to changes in position, for modeling responses to hemorrhage, as a technique for evaluating of orthostatic hypotension, as a method to study hemodynamic and neuroendocrine responses in congestive heart failure, autonomic dysfunction and hypertension, as well as a tool for drug research [7,10-14]. It also has become a useful device in the mobilization of spinal cord and traumatic brain injured patients, as well as in patients suffering from stroke [15]. The key feature of a tilt table is the continuously adjustable position of a patient from horizontal to vertical. This represents an orthostatic challenge, because blood pools in the lower extremities, with the danger that in susceptible individuals vasovagal syncope could occur within approximately 20 minutes. The afferent end of this reflex pathway may be mediated by left ventricular or right atrium mechanoreceptors that are activated during vigorous contraction around under-filled chambers, in a situation similar to severe hemorrhage. Information from these mechano-receptors travels along vagal afferent C fibers to the brainstem, which mediates the efferent response consisting of withdrawal of sympathetic vasomotor tone and by the vagal system [16,17].

In addition to the traditional tilt table, a novel apparatus with stepping device (tilt stepper) was developed in 1998 at the research department of the Paraplegia Centre of the Balgrist University Hospital in Zurich, Switzerland in collaboration with the Department Orthopaedic II of the Orthopaedic Hospital of Heidelberg to enable a mobilization with stabilized circulation and to begin with a locomotion training in an early state of rehabilitation.

**Figure 1:**
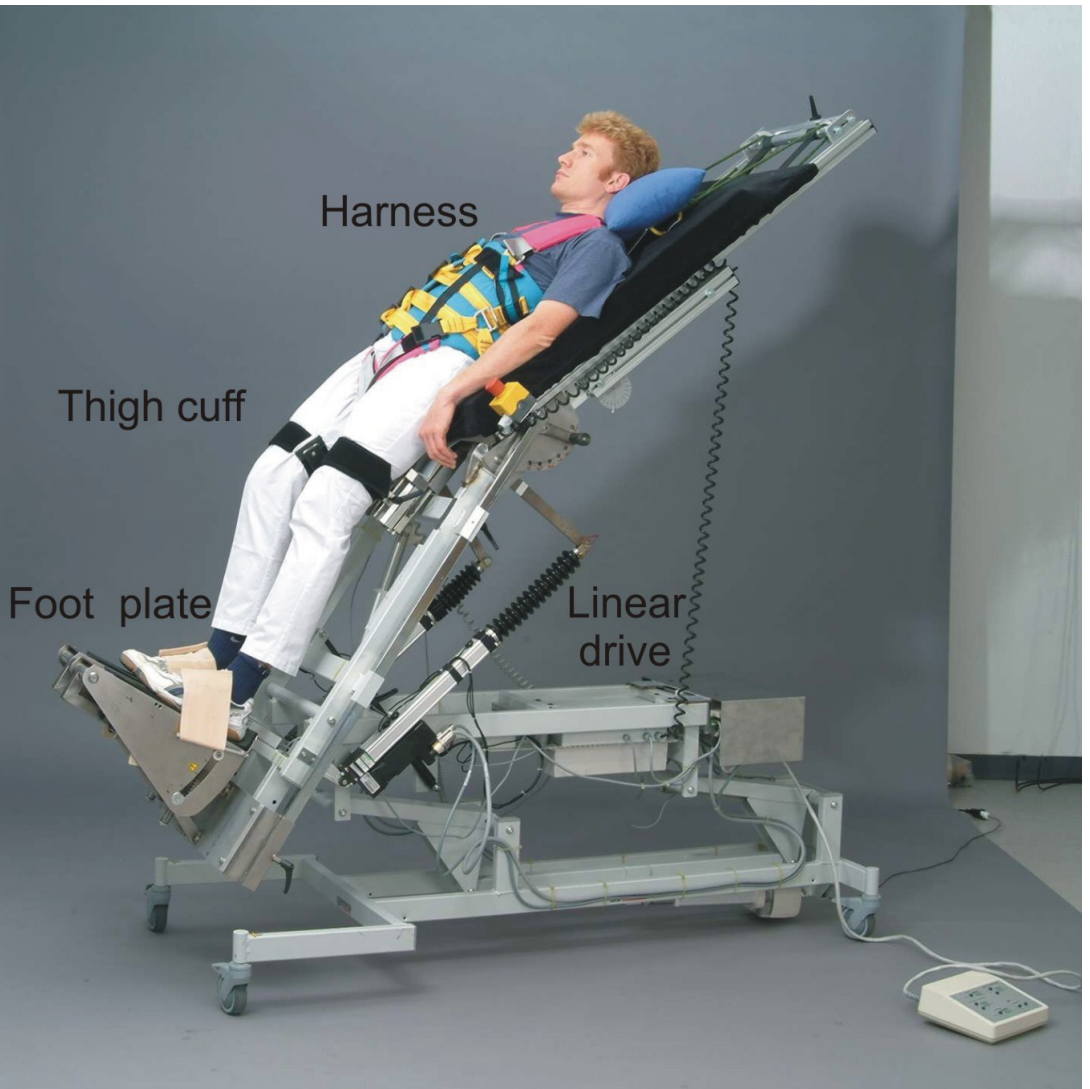
The tilt table with stepping device (tilt stepper)

**Figure 3:**
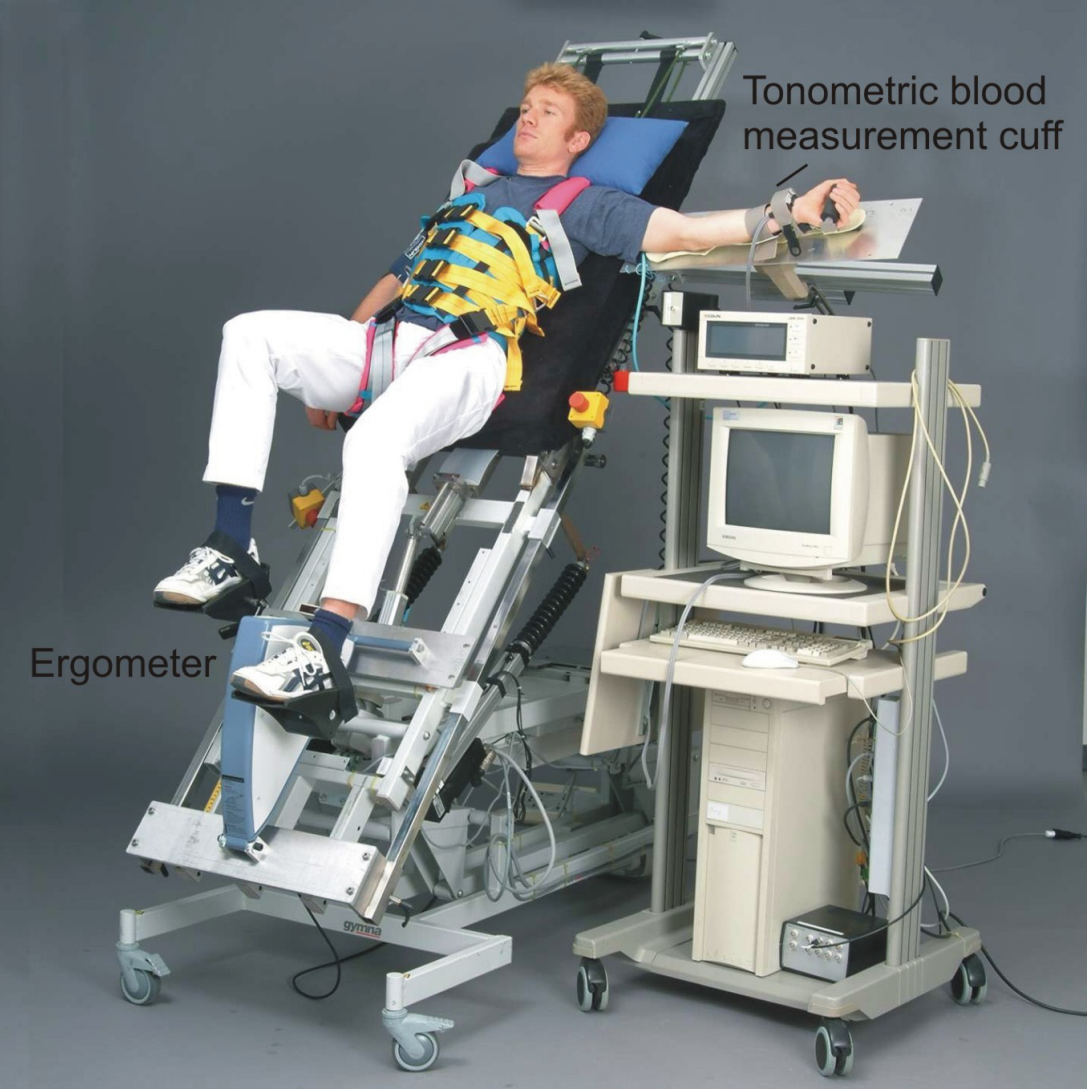
The tilt table with ergometer device (tilt ergometer)

In the tilt stepper, the patient is strapped by a safety belt to the tilt table while the legs are moved passively in a physiological stepping pattern (Figure 1). The inclination can be continuously adjusted from a horizontal to a vertical position. The distribution of the blood correlates directly to the sinus of the angle of inclination. Between 30 and 60 degrees this angle is linear [18]. For inclines larger than 60 degrees, there is a plateau of hemodynamic effects. For the present study, we choose an angle of 75 degrees because in previous studies it has been shown that syncope was more likely to occur at an angle over 60 degrees [18]. To investigate if there is a difference between passive stepping and passive cycling leg movements, we also used a tilt table with an ergometer device (tilt ergometer, Figure 3).

There are only a few studies that have investigated how passive movement of the legs during a tilt table treatment affects circulation. In these studies, either functional electrical stimulation of the leg muscles [19-21] was used, or patients were placed in sitting positions on a cycle ergometer [22]. The results of these studies suggest that passive movements of the legs could stabilize blood circulation. There have also been studies which have utilized a tilt table with passively moving legs. However, in these studies only patients with recurrent vasovagal syncopes were enrolled, the syncope was pharmacologically provoked [5,23-30].

The aim of our experiments was to investigate if passive stepping and cycling movements of the legs during a tilt table test can stabilize blood circulation and prevent neurally mediated syncope in healthy young adults.

## Methods

### Participants

With the permission from the local Ethics Committee and the informed consent of the volunteers, the response of the blood circulation was analyzed in healthy subjects. The exclusion criteria included: recurrent syncope or near-syncope in clinical history, regular medication, abuse of nicotine or alcohol, cardiovascular or neurological diseases, acute or chronic infections, psychiatric disorder and body mass index <18 or >25. All subjects underwent a physical investigation and an ECG was completed one week before the experiment.

In the first experiment, we examined 12 healthy young adults (age 24 ± 5 years) on a traditional tilt table. The subjects, who had syncope or near-syncope were treated on the tilt stepper after a waiting period of 4 weeks. Syncope was defined as a transient loss of consciousness associated with a loss of postural tone. Near-syncope was defined as the appearance of pallor, nausea, light-headedness, diaphoresis or blurred vision. Both conditions were associated with the following hemodynamic changes: a decrease in systolic blood pressure > 60% from baseline values or an absolute value < 80 mmHg (vasodepressor response) and/or a decrease in heart rate > 30 % form the baseline value or an absolute value < 40 beats/min (cardio-inhibitory response) [31].

In the second experiment, we enrolled 42 healthy subjects (age 27 ± 4 years). They were randomized into two groups: group I (23 subjects) was put on a traditional tilt table, while the Group II (19 subjects) on a tilt ergometer. The age of the subjects was restricted to below 35 years, because the cardiovascular response is strongly dependent on age [32].

### Procedures

The aim of the first experiment was to investigate if the blood circulation could be stabilized in people who have a disposition for an "early" appearance of a neurally-mediated syncope on a traditional tilt table. The appearance of a neurally-mediated syncope is physiological and it may occur in all subjects. The interpersonal difference lies in the duration that the subject can be in standing posture until syncope or near-syncope occurs. A decrease of the systolic blood pressure up to maximal 15 mmHg and/or an increase in heart rate up to 20 bpm during the first 6 minutes are considered as a normal reaction to compensate the change in the position of the body [31].

The blood pressure was non-invasively measured with a tonometric blood pressure device. Subjects who suffered a syncope or near-syncope during the first session on the traditional tilt table were in the second session treated on the tilt stepper. In the second experiment, we investigated the effect of passively induced movements on circulation by a cycle ergometer on a tilt table. We enrolled 42 subjects: 23 on a traditional tilt table and 19 on the tilt ergometer.

In both experiments, after 15 minutes of rest the subjects were tilted head-upright at a 75° angle and were returned to the supine position if a syncope or near-syncope occurred or after completion at 30 minutes. Heart rate and blood pressure were measured continuously and non-invasively. Head-up tilt tests were performed in the morning in a dim room. All subjects were instructed to fast overnight and relax the muscles of their lower limbs during the trials. This was monitored with an EMG-measurement on legs (Mm. biceps femoris, rectus femoris, gastrocnemii, tibialis ant.), randomly tested in the first experiment and regularly tested during the second experiment. The EMG signals were amplified and transferred to a personal computer. They were recorded by a data acquisition tool (SolEasy by Aleasolution GmbH, Zurich, CH).

### Tilt table with stepping device (Tilt stepper)

**Figure 2:**
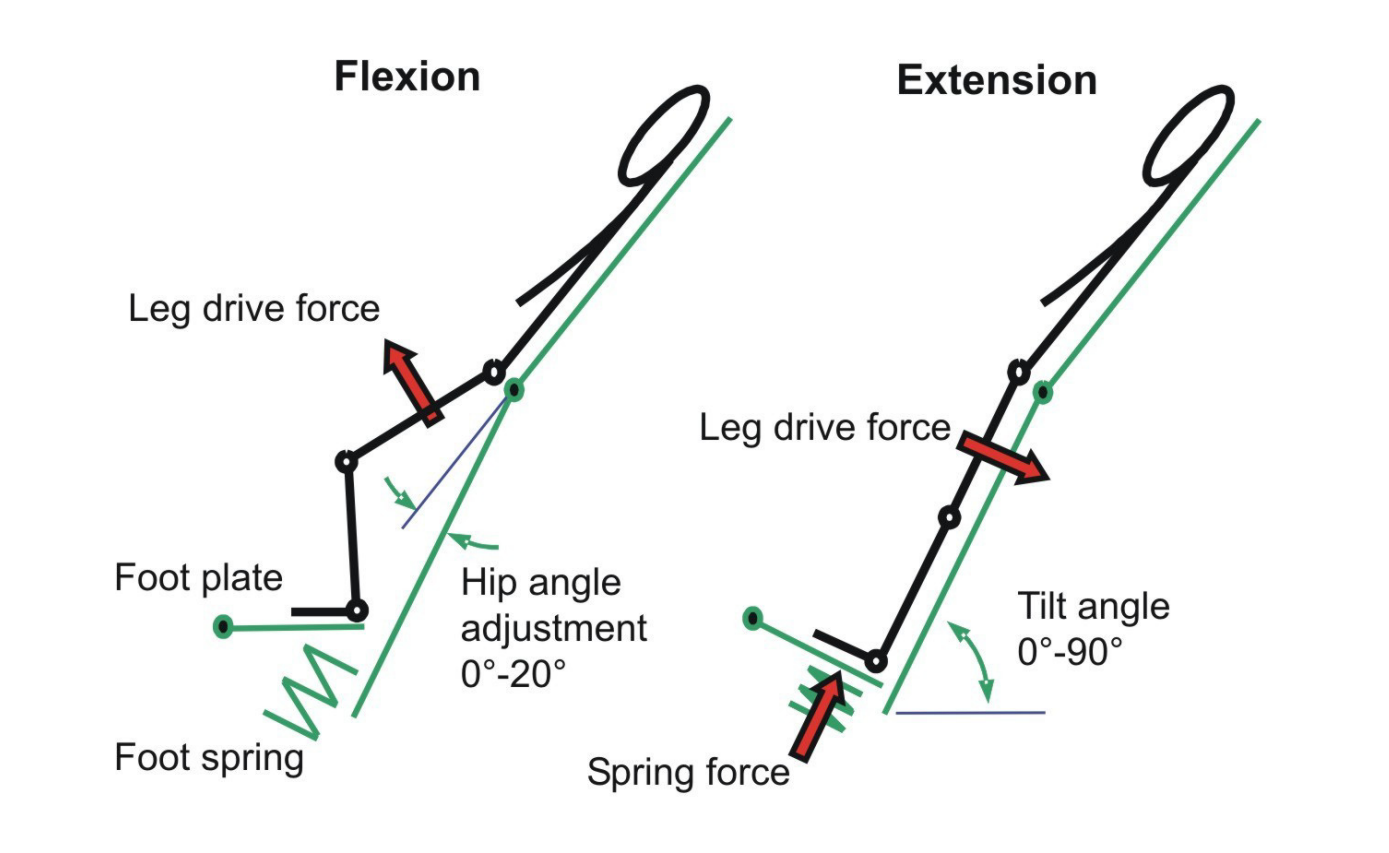
The tilt stepper: generation of leg movements

The tilt stepper is a traditional tilt table (Gymna, Belgium) combined with an integrated leg drive that allows a passive movement of the lower extremities (Figures 1 and 2)

The leg drive that is connected to the thigh by a cuff induces a hip flexion or extension movement. As the feet of the patient are fixed to footplates, the knee is also flexed or extended, respectively. In those phases where the hip and knee joints are extended, the leg pushes down a spring-dampened footplate, which is then again pushed against a foot spring that is mounted within these plates. This footplate generates a loading force on the foot sole of the patient during extension. Applying this cycle of flexion and extension in an alternating way leads to physiological kinetics of the generated motion. A special mechanism is mounted under the hip joint and allows for adjustment of hip extension up to 20°.

Depending on the blood circulation condition of the patient, the device can be tilted to different angles up to a vertical position. This makes it possible for the patient to become accustomed, step-by-step, to the upright position in combination with passive leg movements. The speed of the alternating stepping movements and the range of motion of hip/knee joints can be adjusted by a control panel. The basic construction consists of a linear drive (Parker-Hannifin, Germany), with a precision ball screw that is driven by a synchronous motor via toothed belt (maximum speed 450 mm/sec, maximum force 1400 N, maximum torque of 400 Nm at the hip joint). The movement frequencies range from 0.2 to 0.5 Hz (i.e. one cycle of flexion and extension takes between 2 and 5 sec).

To secure subjects on the tilt table during experiments, fixation with a special harness was used during all experiments (Figure 1).

### The tilt table with ergometer (Tilt ergometer)

The tilt ergometer consists of a traditional tilt table with an additional ergometer device (Tera Joy Germany) that allows a passive cycling movement of the lower extremities. From a technical point of view, the tilt ergometer construction is simpler than the tilt stepper, but it generates a non-physiological motion concerning gait phase related forces on the foot sole. The cycle frequency was between 0.2–0.5 Hz.

### Recordings and Measurement

Blood pressure was measured continuously and non-invasively by a Colin CBM-7000 (Hayashi, Komaki City, Japan). The Colin CBM – 7000 is a tonometric blood pressure device that allows measuring beat-to-beat blood pressure (systolic, mean, diastolic), continuous arterial blood pressure waveform, beat-to-beat and continuous electrocardiography.

### Statistical analysis

In both experiments we used the chi-square test to compare the occurrence of near-syncope/syncope in both groups (tilt table/tilt stepper and tilt table/tilt ergometer). We performed 2 by 4 repeated measures ANOVAs with 2 between factors (device group – namely tilt table vs. tilt stepper or tilt table vs. tilt ergometer), 4 within factors (time – supine, 2 minutes, 6 minutes and end of head-up tilt) and in their interaction (groups × time) for blood pressure and heart rate. Pairwise comparisons were made with the t-test with additional Bonferroni's correction.

## Results

In the first experiment, 7 of 12 subjects (58%) had a syncope or near-syncope on the traditional tilt table. There was an obvious increase in heart rate in the first 6 minutes after changing the position from supine to upright. None of these 7 subjects had a syncope or near-syncope during the treatment session on the tilt stepper 4 weeks later. Comparing the occurrence of near syncope/syncope in both sessions with the chi-square test, there was a significant difference ((χ^2^ (1.1) = 6.465, p = 0.011). Table 1 gives a short overview of these results. The same subjects, who collapsed on the traditional tilt table, did not have syncope or near-syncope while treated on the tilt stepper.

**Table 1:** Occurrence of near-syncope and syncope in experiment one (tilt stepper)

	**no syncope**	**near-syncope**	**syncope**
traditional tilt table [n = 12]	5 (42%)	5 (42%)	2 (18%)
tilt stepper [n = 7]	7 (100%)	0 (0%)	0 (0%)

In the ANOVA for repeated measures there were no significant differences for blood pressure within each group (time 4 levels: supine, 2, 6 and end of head-up tilt; F (1,6) = 4.66, p < 0.0743), but there were significant differences between groups (two levels: tilt stepper and tilt table; F (3,33) = 6.33, p < 0.0016)) and in the interactions (F(3,18) = 7.24, p < 0.0022). The blood pressure differs between the two treatments at the end of head – up tilt (p < 0.0029), but not at 2 minutes (p < 1.000) and at 6 minutes (p < 1.000) (Pairwise comparisons with the t-test and additional Bonferroni's correction). However, there could be shown a trend for a higher blood pressure at 2 minutes and at 6 minutes after head-up tilt in the group treated in the tilt stepper.

There were significant differences for heart rate within each group (time 4 levels: supine, 2, 6 and end of head-up tilt; F (1, 6) = 12.17, p < 0.0130) between groups (two levels; F (3, 33) = 21.16, p < 0.0001) and in the interactions (F (3, 18) = 8.68, p < 0.0009). For the group treated on the traditional tilt table, pairwise comparisons with the t-test with additional Bonferroni's correction showed a significantly higher heart rate at 2 minutes (p < 0.0.0060), but no significant differences at 6 minutes (p < 0.2051) and at the end of head-up tilt (p < 1.000).

In the second experiment, 13 of 23 subjects (57 %) who were on the traditional tilt table had syncope (3) or near-syncope (10). None of the 19 subjects who were on the tilt ergometer had syncope but 4 subjects had near-syncope (21%). Comparing the occurrence of near syncope/syncope in both sessions with the chi-square test (χ^2^ (1.1) = 5.443) there was a significant difference (p = 0.021) (Table 2).

**Table 2:** Mean blood pressure and heart Rate +/- SE during 75° head-up tilt on the tilt-stepper

	**during supine position**	**2-min after head-up tilt**	**6-min after head up tilt**	**end of head-up tilt**
**mean blood pressure [mmHg]**				
traditional tilt table [n = 12]	90 +/- 4	95 +/- 4	94 +/- 4	80 +/- 3*
tilt stepper [n = 7]	89 +/- 4	93 +/- 5	97 +/- 2	95 +/- 6*
**heart rate [beats/min]**				

traditional tilt table [n = 12]	65 +/- 5	80 +/- 5*	78 +/- 4	65 +/- 5
tilt stepper [n = 7]	61 +/- 3	69 +/- 3*	71 +/- 3	71 +/- 5

* p < 0.05 (compared with ANOVA for repeated measures)

In the ANOVA for repeated measures, there were significant differences for blood pressure within each group (time 4 levels: supine, 2, 6 and end of head-up tilt; F (1,6) = 34.43, p < 0.0001) between groups (two levels; F (3,33) = 13.42, p < 0.0001)) and in the interactions (F(3,18) = 10.95, p < 0.0001). Pairwise comparisons with the t-test (additional Bonferroni's correction) showed no significant differences at 2 minutes (p < 0.5221) and at 6 minutes (p < 0.4429) but a significant difference at the end of head – up tilt (p < 0.0001). However, there could be shown a trend for a higher blood pressure at 2 minutes and at 6 minutes after head-up tilt in the group treated on the tilt ergometer. There were significant differences for heart rate within each group (time 4 levels: supine, 2, 6 and end of head-up tilt; F (1,6) = 12.17, p < 0.0130), between groups (two levels; F (3,33) = 21.16, p < 0.0001), and in the interactions (F(3,18) = 8.68, p < 0.0009). Pairwise comparisons with the t-test (additional Bonferroni's correction) showed no significant differences at 2 minutes (p < 0.3317), but a significantly higher heart rate in the group treated on the tilt table at 6 minutes (p < 0.0007) and at the end of head – up tilt (p < 0.0002).

All subjects on the tilt stepper and tilt ergometer completed 30 minutes of head-up tilt. The duration of the head-up tilt was different in the group on the traditional tilt table, as an abrupt decrease of blood pressure or symptoms of near-syncope occurred.

In the head-up tilt position the subject stands on the footplates on the tilt stepper, whereas in the tilt ergometer the harness holds the whole body weight. The subjects who were investigated on the tilt stepper felt comfortable during the whole experiment, whereas the subjects examined on the tilt ergometer in experiment two complained of discomfort. The subjects on the tilt ergometer experienced more discomfort because of the perception of no lower limb support. These statements were subjective; no standardized assessment instrument was used to measure the comfort.

**Figure 4:**
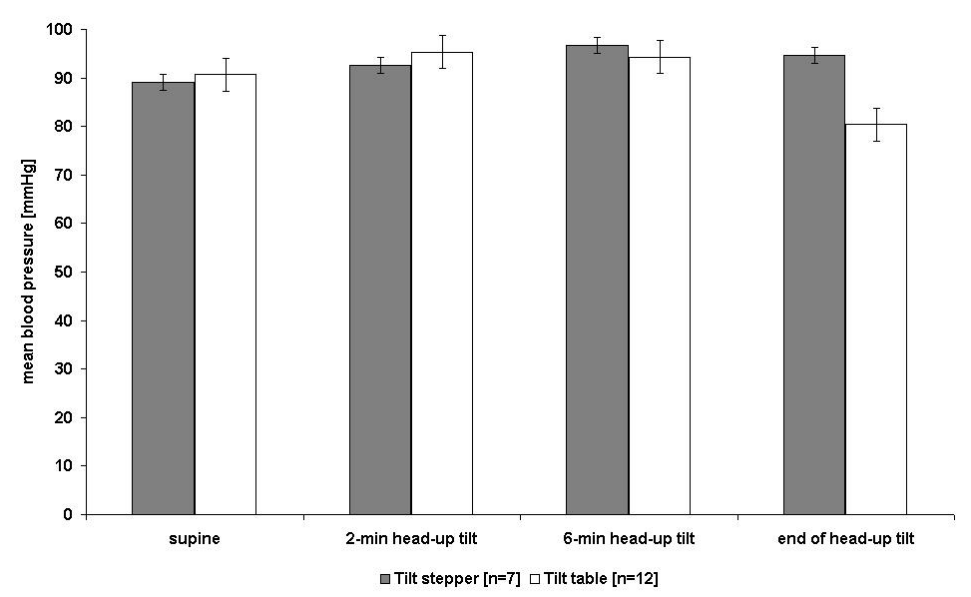
Blood pressure +/- SE during 75° the tilt table and tilt stepper test

**Figure 5:**
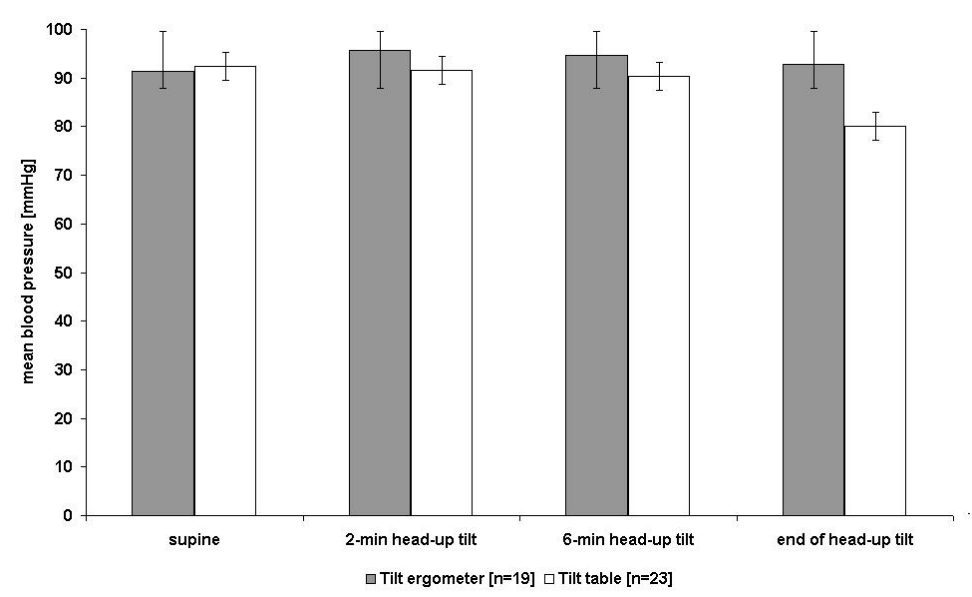
Blood pressure +/- SE during 75° the tilt table and tilt ergometer test

Tables 3 and 4 and Figures 4 and 5 provide an overview about the response of the blood pressure of subjects tested on the traditional tilt table (with and without syncope, n = 12 in experiment one and n = 23 in experiment two) and subjects with passive leg movements during the tilt table test on the tilt stepper (n = 7) and tilt ergometer n = 19).

**Table 3:** Occurrence of near-syncope and syncope in experiment two (tilt ergometer)

	**no syncope**	**syncope**	**near-syncope**
traditional tilt table [n = 23]	10 (43%)	3(14%)	10 (43%)
tilt ergometer [n = 19]	15 (79%)	0 (0%)	4 (21%)

**Table 4:** Mean blood pressure and heart rate +/- SE during 75° head-up tilt on the tilt ergometer

	**during supine position**	**2-min after head-up tilt**	**6-min after head-up tilt**	**end of head-up tilt**
**mean blood pressure [mmHg]**				

traditional tilt table [n = 23]	92 +/- 4	92 +/- 6	90 +/- 5	80 +/- 4*
tilt ergometer [n = 19]	91 +/- 5	96 +/- 3	95 +/- 2	93 +/- 4*
**heart rate [beats/min**]				

traditional tilt table [n = 23]	64 +/- 5	79 +/- 5	82 +/- 3*	78 +/- 5*
tilt ergometer [n = 19]	65 +/- 3	74 +/- 4	73 +/- 5*	68 +/- 4*

* p < 0.05 (compared with ANOVA for repeated measures)

**Figure 6:**
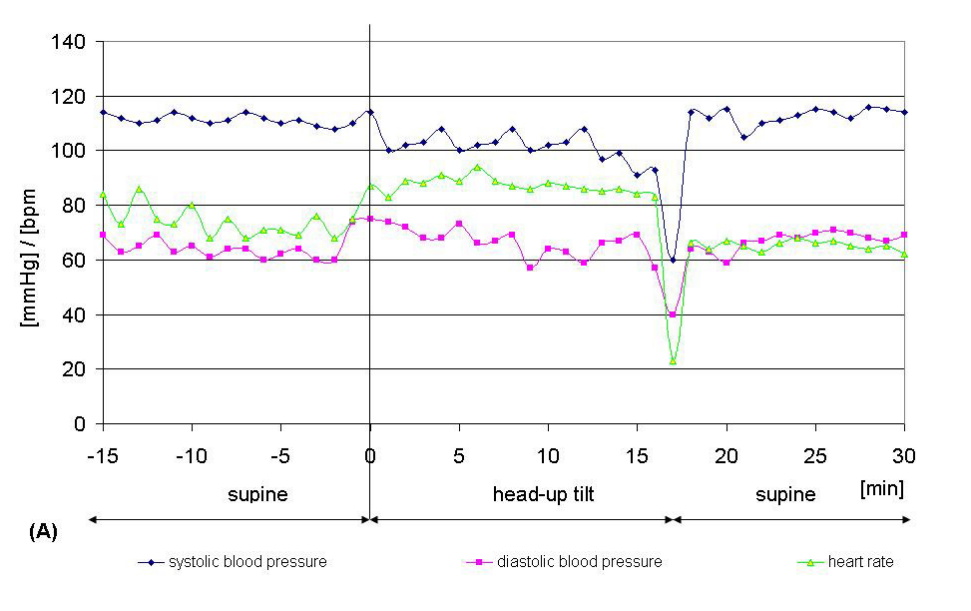
Typical recordings illustrating a subject with syncope. RF = M. rectus femoris, BF = M. biceps femoris, TA = M. tibialis anterior, GM = M. gastrocnemius

**Figure 7:**
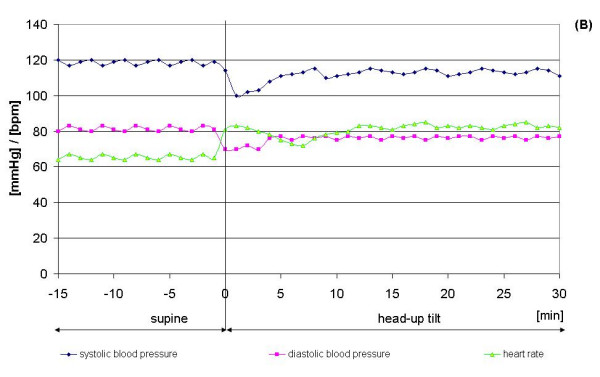
Typical recordings illustrating a subject without syncope. RF = M. rectus femoris, BF = M. biceps femoris, TA = M. tibialis anterior, GM = M. gastrocnemius

In Figures 6 and 7, recordings illustrating the development of systolic and diastolic blood pressure and heart rate for one subject with syncope (Figure 6) and another subject without syncope (Figure 7) during the tilt table test are shown. The observed progression of blood pressure and heart rate of the subject who had syncope is typical for a neurally-mediated syncope, because of the sudden decrease of systolic and diastolic blood pressure combined with bradycardia more than 20 minutes after head-up tilt. Also typical is the increase in heart rate observed in the first 6 minutes after head-up tilt. All subjects treated on the tilt table had this benign form of syncope and showed a similar blood pressure and heart rate progression during the tilt table test.

Figure 7 is a good example for the normal progression of blood pressure and heart rate during a tilt table test. 2 minutes after head-up tilt there is a slight decrease of systolic and diastolic blood pressure and a slight increase of heart rate, a physiological mechanism of compensation for the change of position (supine to head-up tilt).

**Figure 8:**
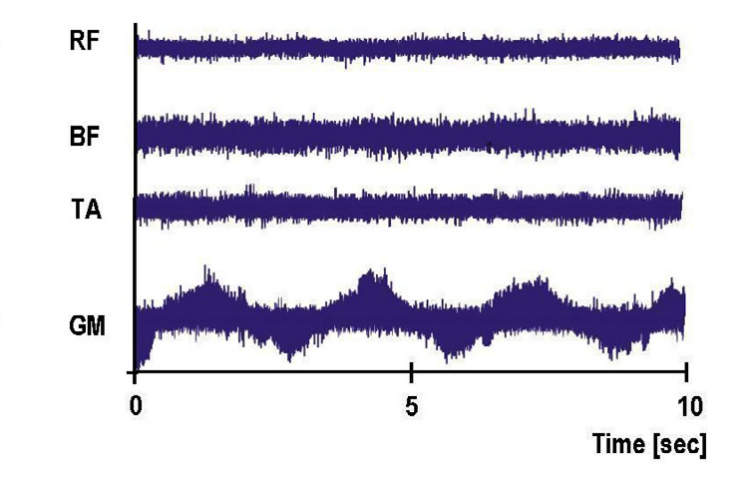
Muscle activity during the tilt table and tilt stepper test

**Figure 9:**
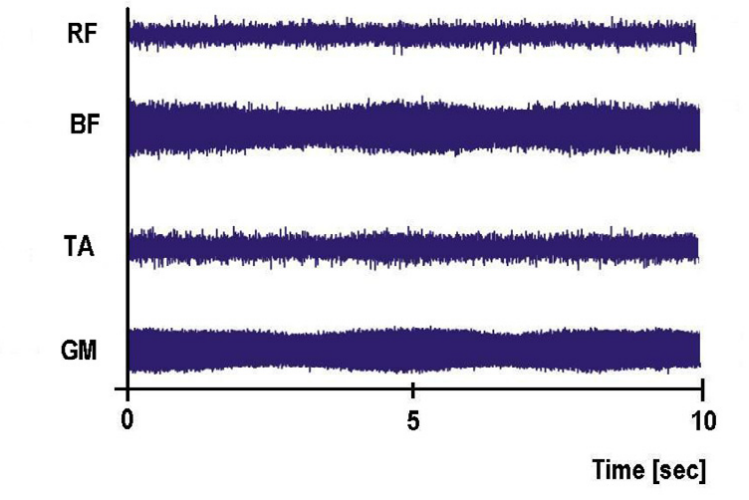
Muscle activity during the tilt table and tilt stepper test

Figure 8 is an example for the EMG activity in the right leg during the tilt stepper test, and Figure 9 during the tilt table test. It becomes obvious that there is no active muscle activity. The ups and downs in the curve of the muscle gastrocnemius on the tilt stepper are from the passive movements.

## Discussion

The tilt table is an apparatus that has become an important part in the evaluation of patients with unexplained syncope or loss of consciousness [14,24,33]. It has also proven useful for circulatory training of patients suffering from several neurological diseases. However, the treatment is limited by the occurrence of circulatory collapse [16]. Both hypotension and bradicardia leading to syncope during tilt tests are also common events in healthy persons. These responses are considered to be part of a reflex response triggered by a sympathetic-induced hypercontraction of an almost empty left ventricular chamber [34]. In both experiments there were no recurrent syncope or near-syncope in the clinical history of the subjects and the ECG did not show any abnormities. For these reasons, and because of the development of the heart rate and blood pressure in our experiments, the occurring syncopes and near-syncopes that occurred ought to be benign and so called neurally-mediated syncopes or vasovagal syncopes. It is a physiological form of syncope that can occur in healthy persons. Some persons have the disposition of suffering a neurally-mediated syncope earlier than others [35]. This benign form of syncope can be differentiated from malignant syncopes, like the hyperadrenergic orthostatic hypotension (decrease of blood pressure and increase of heart rate), hypoadrenergic orthostatic hypotension (decrease of blood pressure without an increase of heart rate) and postural tachycardia syndrome (massive increase of heart rate without decrease in blood pressure) by recording heart rate and blood pressure [31].

Although the tilt table has become an accepted diagnostic tool, there are no comparable studies with the tilt table in which the effect of passive leg motion on circulation have been investigated.

The aim of these two experiments was to investigate if passive leg movements during head-up tilt can prevent syncope. The data in the present study show a stabilizing effect on the blood circulation and this study suggests that there is an effect on preventing neurally-mediated syncope by both devices. In the first experiment, none of the subjects who had syncope/near-syncope on the traditional tilt table had syncope/near-syncope four weeks later on the tilt stepper. In the second experiment, only 4 subjects who were treated on the tilt ergometer had near-syncope. In both experiments the increase of heart rate was larger in the group tested on the traditional tilt table. A correlation between heart rate and appearance of syncope was described [16,36]. An increase in heart rate > 18 bpm during the first minutes after changing position from supine to upright leads to syncope, with a sensitivity of 90% and a specification of 100%. Consequently, the positive effect of passive leg movement on heart rate is obvious. Heart rate and blood pressure give an indication of the sympathetic activity, which is activated on the tilt table [37,38]. This increased sympathetic activity stimulates mechano-receptors in the ventricle, which leads to an activation of the vagus nerve and a reflexive decrease of sympathetic activity. The vagus activity leads to bradycardia and vasodilatation: the Bezold-Jarisch-Reflex [36]. We suggest that the sympathetic activity becomes reduced by the tilt stepper, preventing this vicious cycle that leads to a vasovagal syncope. This has to be proved in further studies by an intra-arterial catecholamine measurement.

In the first experiment we treated the same subject twice on a tilt table. It cannot be excluded that an adaptation to the orthostatic change occurred in these subjects. However, there was an interval of four weeks between the first treatment on the traditional tilt table and the second treatment on the tilt stepper. Therefore, a training effect or an effect of habituation, such as described in another study in which patients suffering from syncope were treated each day over 6 weeks, seems to be unrealistic [39].

The results in both experiments indicate that blood circulation can be stabilized by passive leg movements. However, the movements of the two devices used in these experiments are very different: on the tilt stepper there are stepping like movements and the legs can be loaded during extension and unloaded in flexion. In the tilt ergometer, the movements are the other way round. There might be more afferent input from the load receptors in the tilt stepper compared to the tilt ergometer. For example it could be shown that the load moments acting about the bilateral hip, knee and ankle joint axes during cycling are found to be generally lower than those induced during normal level walking [10] and concluded that afferent input from hip joints, in combination with that from load receptors during walking, plays a crucial role in the generation of locomotor activity in the isolated human spinal cord [1]. Also, the range of motion is adjustable in the tilt stepper, so that the extent of flexion and extension can be increased or decreased depending on the condition of the patient. Therefore, the tilt stepper may be more effective in activating a locomotion pattern. In addition, both devices might help to decrease spasticity [40] and serve to prevent osteoporosis [41]. Although these effects were not part of our current investigation, some of these issues could be proven in trials with treadmill training in the rehabilitation of patients with stroke, spinal cord and traumatic brain injury [18,39]. Thus, we plan to use the tilt stepper in further studies to investigate if it leads to a stabilization of blood circulation, prevention of neurally mediated syncope in an early state of rehabilitation, decrease in spasticity, prophylaxis of osteoporosis and activation of the locomotion pattern generator of patients suffering from neurological diseases. This in turn may lead to a better outcome and quality of life for the patient.

In conclusion, we could show that both passive cycle and stepping movements of the legs during head-up tilt testing can stabilize blood circulation and prevent syncope in young healthy people. In further studies, we aim to investigate if the tilt stepper could become a helpful device for patients suffering from neurological diseases.
